# Assessment of the Relationship Between Red Cell Distribution Width and Multiple Sclerosis

**DOI:** 10.1097/MD.0000000000001182

**Published:** 2015-07-24

**Authors:** You-Fan Peng, Wen-Yan Cao, Qiong Zhang, Dan Chen, Zhao-Xia Zhang

**Affiliations:** From the Laboratory Medicine Diagnostic Center (Y-FP, W-YC, QZ, Z-XZ); and Department of General Medicine, The First Affiliated Hospital, Xin Jiang Medical University, Xinjiang Urumqi, China (DC).

## Abstract

Accumulating data have shown that immune and inflammatory factors are involved in the pathogenesis of multiple sclerosis (MS), and loss of polyunsaturated fatty acids from plasma and blood cell membranes has also been reported in patients with MS, contributing to the variation of erythrocyte deformability. Therefore, the aim of this investigation was to assess the association between red blood cell distribution width (RDW) and MS.

A total of 109 patients with MS and 130 healthy individuals were enrolled into the study, and MS patients receiving treatment of subcutaneous recombinant Rebif (IFN-β1a) were followed retrospectively. Complete blood cell counts and Expanded Disability Status Scale (EDSS) score were evaluated in patients with MS before and after treatment.

RDW values were significantly higher in patients with MS compared with the controls (13.6 ± 0.89 vs 12.8 ± 0.38, *P* *<* 0.001); a positive correlation between RDW and EDSS score was observed in patients with MS (*r* = 0.789, *P* < 0.001). Significant differences in the value of RDW and EDSS score were observed between treatment-naive patients and treated patients (13.6 ± 0.95 vs 12.7 ± 0.44, *P* < 0.001; 3.6 ± 1.39 vs 1.5 ± 0.60, *P* < 0.001). RDW was associated independently with MS in logistic regression analysis (odds ratio = 7.007; 95% confidence interval [CI] 3.461–14.187; *P* < 0.001), and receiver-operating characteristics (ROC) analysis showed that a RDW measurement >13.11% evaluated MS with a sensitivity of 70.0% and a specificity of 84.7%, and the area under the ROC curve for RDW was calculated as 0.80 (95% CI 0.739–0.859, *P* < 0.001). The level of RDW was decreased in treatment responders with the reduction of EDSS score; a strong relationship was also observed in treatment responders between RDW and EDSS score (*r* = 0.733, *P* < 0.001), and covariance analysis indicated RDW values decreased significantly in treatment responders (*P* = 0.025).

Our results suggest that elevated RDW values are associated with EDSS score in patients with MS, and the relationship is remarkably influenced by Rebif treatment; RDW may be a useful marker to estimate disability status and treatment effectiveness in patients with MS.

## INTRODUCTION

Multiple sclerosis (MS) is an autoimmune disease of the nervous system characterized by the damage of myelin sheath around nerve fibers, leading to a variety of neurological disorders including dysfunction of the brain stem, cerebellum, spinal cord, and optic nerve.^1^ Accumulated data have indicated that immune and inflammatory factors are involved in the pathogenesis of MS.^2^ Recently, a line of evidence also attests that antioxidant treatment is effective in patients with MS.^3^

Red blood cell distribution width (RDW) is an objective measured value, which reflects the variability of circulating red blood cells (RBCs) and is used for the differential diagnosis of anemia. In the past several years, RDW has received the attention in the field of inflammation inasmuch as it was associated with the presences and outcomes in patients with polycystic ovary syndrome, ankylosing spondylitis, myocardial infarction, acute pancreatitis-associated lung injury, and spontaneous echo contrast.^4–8^ Magnetic resonance imaging (MRI), as a expensive checking method, is clinically a valuation methodology of brain or spinal cord disease activity of MS.^9^ It has been previously observed that the pathogenesis of MS is associated with inflammatory factors, and found that pro-inflammatory cytokines increase in patients with MS such as tumor necrosis factor (TNF) and interleukin-1β (IL-1β).^10–11^ It is then noteworthy that loss of polyunsaturated fatty acids from plasma and blood cell membranes is observed in patients with MS,^12^ which contributes to the variation of erythrocyte deformability. Therefore, the aim of this investigation was to assess the association between RDW and MS.

## PATIENTS AND METHODS

A total of 109 patients with MS and 130 healthy individuals were enrolled into this study. The research related to human use has been complied with all the relevant national regulations, institutional policies, and in accordance with the tenets of the Helsinki Declaration, and has been approved by The First Affiliated Hospital of Xinjiang Medical University institutional review board. Informed consent has been obtained from individuals included in this study. All patients with clinically active disease on admission (defined as onset of new neurological symptoms or signs attributable to demyelination within the previous 2 weeks) were the relapsing-remitting of MS and defined as MS according to the international criteria.^13^ The Expanded Disability Status Scale (EDSS) score was used to evaluate disease progression of patients with MS.^14^ Exclusion criteria were determined to refuse participants with following diseases: hematopathy, diabetes, hypertension, cardiovascular diseases, liver and kidney dysfunction, malignancies, and other inflammatory diseases that could potentially interfere with the measurement of RDW. Blood samples were tested with respect to leukocyte, lymphocyte, hemoglobin, mean corpuscular volume, platelet, and RDW before and after treatment. The reference range of RDW in our laboratory is 1% to 15.0%.

## STATISTICAL ANALYSIS

SPSS16.0 statistical software was used to analyze the clinical data (SPSS Inc, Chicago, IL). Continuous variables were shown as mean ± standard deviation, and categorical variables were presented as percentage. Kolmogorov–Smirnov test was used to examine whether the data were normally distribution. Student *t* test, *χ*^2^ test, and Mann–Whitney *U* test were used to compare the difference between groups. Correlations between 2 continuous variables were evaluated with Pearson test. Variables found to be significant on univariate analysis were entered into Binary logistic regression analysis. Further, receiver operating characteristics (ROC) curve analysis was used to measure the performance of RDW. An unpaired Student *t* test was then used to test for significant differences between treatment-naive patients and treated patients, and we used paired Student *t* test to compare EDSS score and RDW before and after treatment. A covariance analysis was also performed to control underlying factors before and after treatment. Statistical significance was set at *P* < 0.05.

## RESULTS

**FIGURE 1:**
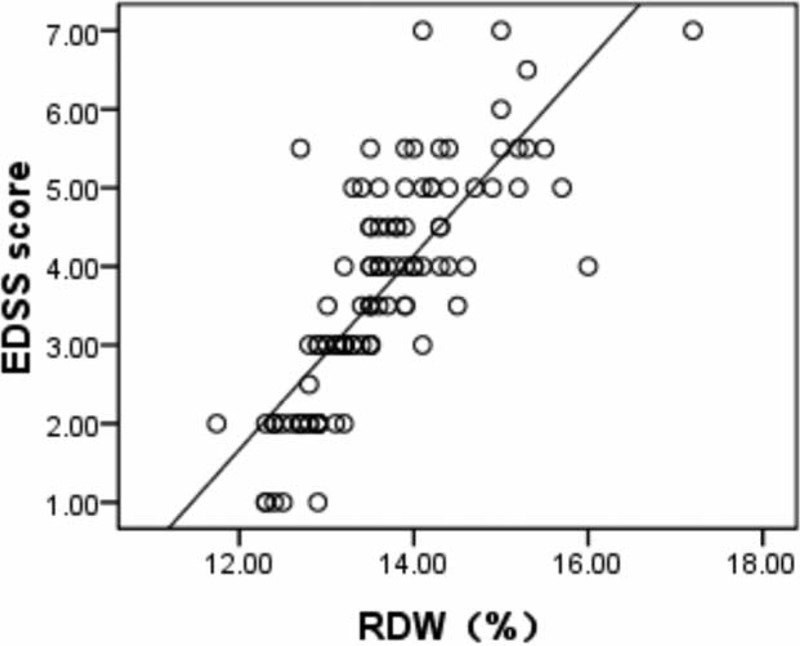
Scatter plot indicating a correlation between RDW and EDSS score (*r* = 0.789, *P* < 0.001) in patients with MS. EDSS = Expanded Disability Status Scale, MS = multiple sclerosis, RDW = red cell distribution width.

**FIGURE 2:**
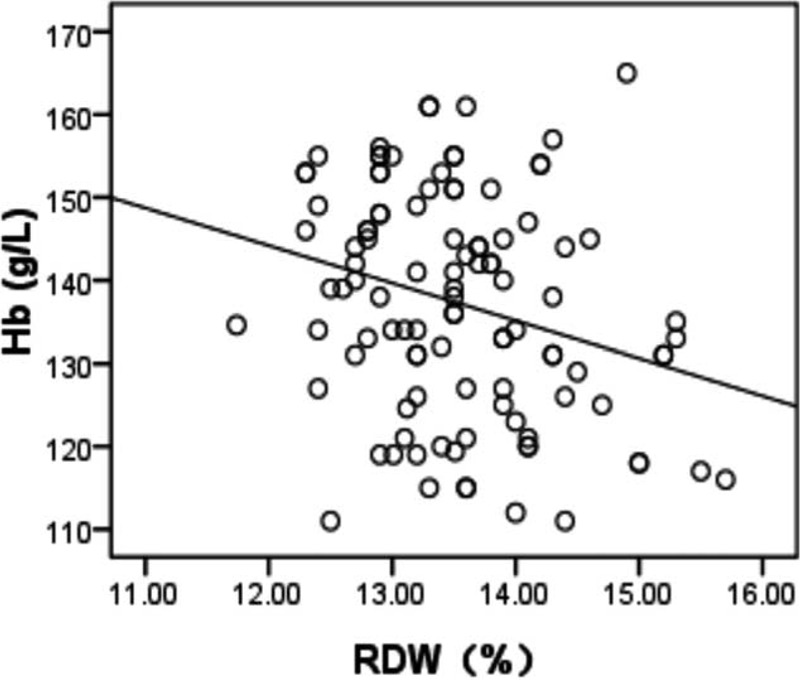
Scatter plot indicating a correlation between RDW and Hb (*r* = −0.386, *P* < 0.001) in patients with MS. Hb = hemoglobin, MS = multiple sclerosis, RDW = red cell distribution width.

Characteristics of all patients and healthy participants are shown in Table 1. RDW values were significantly higher in patients with MS compared with the controls (13.6 ± 0.89 vs 12.8 ± 0.38, *P* < 0.001). Sex, age, leukocyte count, and platelet count were not different between groups. The level of RDW was negative correlated with hemoglobin in patients with MS (*r* = −0.386, *P* < 0.001); of note, a positive correlation between RDW and EDSS score was observed in patients with MS (*r* = 0.789, *P* < 0.001), as shown in Figures 1 and 2. Significant differences in the value of RDW and EDSS score were observed between treatment-naive patients and treated patients in Table 2 (13.6 ± 0.95 vs 12.7 ± 0.44, *P* < 0.001; 3.6 ± 1.39 vs 1.5 ± 0.60, *P* < 0.001).

**TABLE 1:**
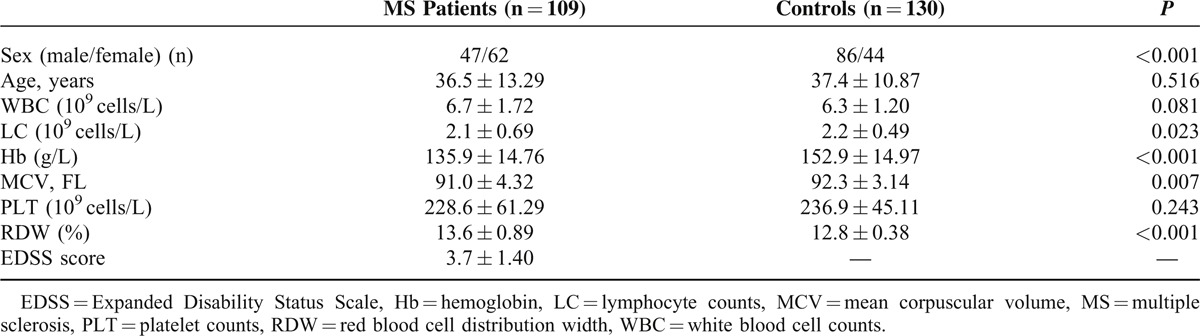
Baseline Characteristics of Patients With MS and Control Groups

**TABLE 2:**
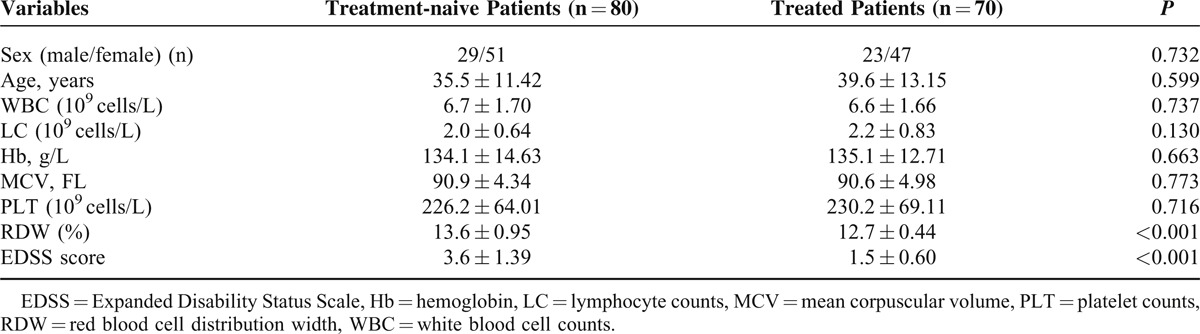
A Comparison Between Treatment-naive Patients and Treated Patients

**FIGURE 3:**
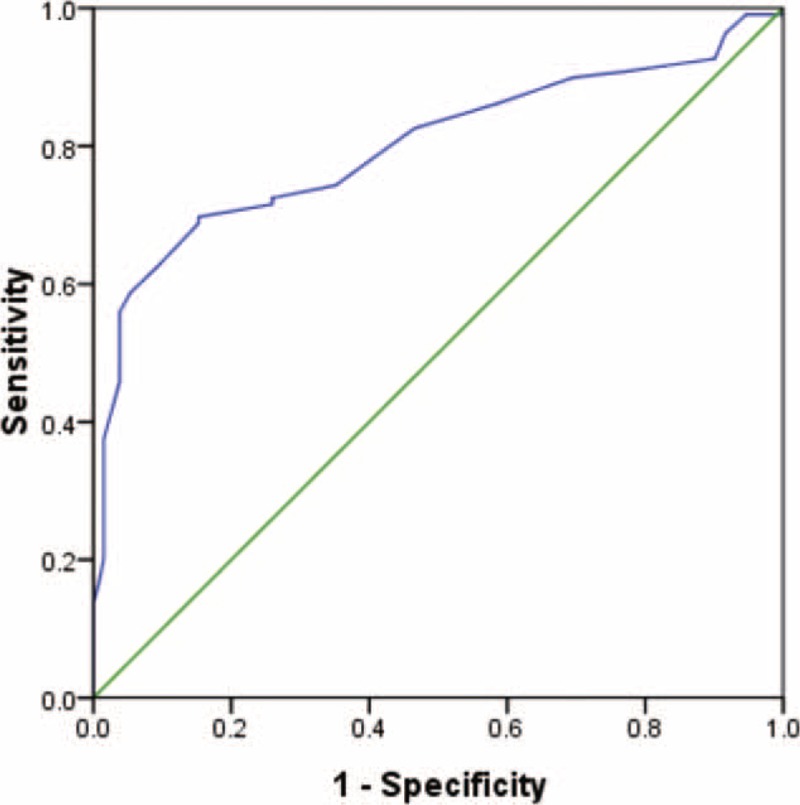
The ROC curve analysis of RDW to estimate patients with MS. RDW measurement >13.11% evaluated MS with a sensitivity of 70.0% and a specificity of 84.7%, and the area under the ROC curve for RDW was calculated as 0.80 (95% CI 0.739–0.859, *P* < 0.001). MS = multiple sclerosis, RDW = red cell distribution width, ROC = receiver-operating characteristics.

RDW was independently associated with MS when Logistic regression analysis was used to identify potential factors associated with MS (odds ratio = 7.007; 95% confidence interval [CI] 3.461–14.187; *P* < 0.001), as shown in Table 3. ROC curve analysis showed that a RDW measurement>13.11% evaluated MS with a sensitivity of 70.0% and a specificity of 84.7%, and the area under the ROC curve for RDW was calculated as 0.80 (95% CI 0.739–0.859, *P* < 0.001) (Figure 3).

**TABLE 3:**
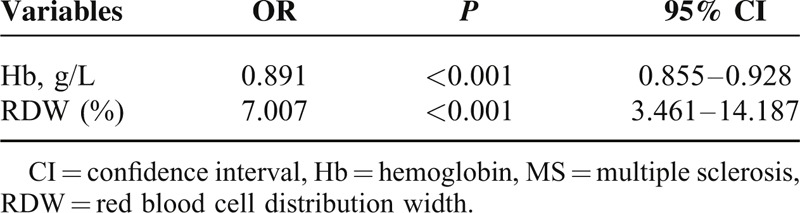
The Potential Factors Associated With MS as Evaluated by Binary Logistic Regression Analysis

Patients who exist infections clinically in the recent were excluded. A total of 39 patients who receive treatment of subcutaneous recombinant Rebif (IFN-β1a) 50 μg 3 times per week were followed retrospectively for an average of 2.3years (2.3 ± 1.15 years) to verify whether RDW was used to estimate the effectiveness of long-term treatment for patients with MS. Our results showed that 39 patients responded for Rebif long-term treatment according to the criterion (patients who advanced ≥1.0 point in EDSS were defined as treatment responders). Complete blood cell counts of treatment responders and their EDSS score were compared before and after treatment, and the results displayed that the level of RDW decreased in treatment responders with the reduction of EDSS score (13.6 ± 0.83 vs 12.5 ± 0.42, *P* < 0.001; 3.5 ± 1.25 vs 1.4 ± 0.61, *P* < 0.001) (Table 4). To assess that Rebif has an effect on RDW in patients with MS, correlation analysis and analysis of covariance (ANCOVA) were used; a strong relationship was observed in treatment responders between RDW and EDSS score (*r* = 0.733, *P* < 0.001), and ANCOVA indicated that RDW values decreased significantly in treatment responders (*P* = 0.025).

**TABLE 4:**
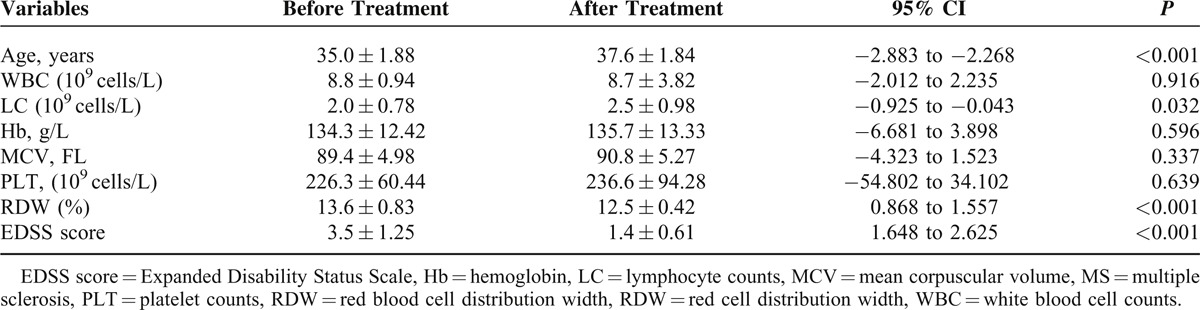
Comparison of Variables Including RDW and EDSS Score in Patients With MS Before and After Treatment

## DISCUSSION

It has been well documented that TNF in the cerebrospinal fluid is associated with disability and nerve injury state in patients with progressed MS.^15^ In the earlier trials, most studies that have researched the correlation between several inflammation indicators and short-term disease progression in MS have shown negative results.^16^ There is mounting evidence that several inflammatory markers are not elevated in patients with MS such as C-reactive protein (CRP) and erythrocyte sedimentation rate.^12,17–18^ In our study, increased RDW values were observed in patients with MS than healthy individuals, and it was associated with EDSS score in patients with MS. Interestingly, the level of RDW was significantly lower in MS patients undergoing treatment Rebif after adjusting potential factors, indicating that RDW might be a useful marker to evaluate treatment effects in patients with MS. It has been demonstrated in previous trials that ratio of lipid to protein decreases in patients with MS,^19^ and inflammatory states can alter membrane lipid composition and membrane fluidity in patients with MS.^12^ In fact, loss of polyunsaturated fatty acids from plasma and blood cell membranes has also been reported in patients with MS,^20^ contributing to influence erythrocyte deformability and membrane fluidity in patients with MS,^12^ whereas erythrocyte deformability and volume main depend on membrane fluidity^21–22^; of note, Patel et al^23^ reported an association between RDW and erythrocyte deformability, and found that decreased erythrocyte deformability increases RDW values. Obviously, a lot of loss of polyunsaturated fatty acids from erythrocyte membranes increases RDW values in patients with MS via altering erythrocyte deformability. In addition, chronic inflammation of central nervous system and immune factors play a critical role in the pathogenesis of MS.^24–25^ Thus, long-term chronic inflammation may be a possibility that explains the association of RDW and MS. Indeed, numerous studies in the literature have reported that inflammation contributes to increase the level of RDW through influencing erythrocyte heterogeneity.^26–28^

We are aware, however, that there may be several limitations to this study. First, our study was limited by a small number, especially in treatment patients. Moreover, other inflammatory markers were not evaluated in patients with MS, such as CRP, TNF, and IL-6. Finally, the detail situation and brain or spinal cord activity of MRI for patients both during and after treatment were not recorded in the present study, as well as the association between RDW and brain or spinal cord activity of MRI should be evaluated in MS patients. However, our results suggest that elevated RDW values are associated with MS, and that the relationship is remarkably influenced by Rebif treatment; RDW, as an inexpensive and available test, may be a useful marker to estimate disability status and treatment effectiveness in patients with MS.
